# The role of transcriptional regulation in maintaining the availability of mycobacterial adenylate cyclases

**DOI:** 10.7717/peerj.298

**Published:** 2014-03-11

**Authors:** Sarah J. Casey, Mica J. Ford, Michaela A. Gazdik

**Affiliations:** 1 Biology Department, Ferrum College, Ferrum, VA, United States; 2 Department of Biomedical Sciences and Pathobiology, VA-MD Regional College of Veterinary Medicine, Blacksburg, VA, United States

**Keywords:** Cyclic adenosine monophosphate, Adenylate cyclase, *Mycobacterium tuberculosis*, Gene regulation, *Mycobacterium smegmatis*

## Abstract

Mycobacterium species have a complex cAMP regulatory network indicated by the high number of adenylate cyclases annotated in their genomes. However the need for a high level of redundancy in adenylate cyclase genes remains unknown. We have used semiquantitiative RT-PCR to examine the expression of eight *Mycobacterium smegmatis* cyclases with orthologs in the human pathogen *Mycobacterium tuberculosis*, where cAMP has recently been shown to be important for virulence. All eight cyclases were transcribed in all environments tested, and only four demonstrated environmental-mediated changes in transcription. *M. smegmatis* genes MSMEG_0545 and MSMEG_4279 were upregulated during starvation conditions while MSMEG_0545 and MSMEG_4924 were downregulated in H_2_O_2_ and MSMEG_3780 was downregulated in low pH and starvation. Promoter fusion constructs containing *M. tuberculosis* H37Rv promoters showed consistent regulation compared to their *M. smegmatis* orthologs. Overall our findings indicate that while low levels of transcriptional regulation occur, regulation at the mRNA level does not play a major role in controlling cellular cyclase availability in a given environment.

## Introduction

Cyclic adenosine monophosphate (cAMP) is an important second messenger produced by adenylate cyclase enzymes which controls a wide range of cellular responses in both prokaryotic and eukaryotic cells (Botsford & Harman, 1992; Peterkofsky et al., 1993; Tang & Hurley, 1998; Baumann, Lange & Soppa, 2007). cAMP signaling is critical for the regulation of virulence genes in several bacterial pathogens such as *Yersinia pestis* and *Pseudomonus aeruginosa*, and recent evidence suggests that cAMP also plays a role in the virulence of *Mycobacterium tuberculosis*, the causative agent of tuberculosis (Rickman et al., 2005; Petersen & Young, 2002; Smith, Wolfgang & Lory, 2004; Agarwal et al., 2009). A bacterially-derived cAMP burst is responsible for increased levels of cAMP in *M. tuberculosis* infected macrophages. The increased cAMP leads to decreased phagosome-lysosome fusion (Kalamidas et al., 2006) and increased production of CREB-mediated TNF*α* (Agarwal et al., 2009). Both physiological responses aid in *M. tuberculosis* survival after macrophage phagocytosis. Deletion of adenylate cyclase Rv0386 causes a loss of the bacterial-derived intramacrophage cAMP, leading to decreased bacterial survival during mouse infection (Agarwal et al., 2009). Additionally, deletion of the cAMP-controlled transcription factor, cAMP Receptor Protein (CRP_Mt_), from the *M. tuberculosis* genome causes attenuation of *M. tuberculosis* in a murine model and reduced bacterial growth rates in vitro and within macrophages (Rickman et al., 2005; Akhter et al., 2008).

The classical model for cAMP regulation in bacteria is based on the well characterized cAMP response in *Escherichia coli* (Botsford & Harman, 1992). *E. coli* contains a single class I adenylate cyclase which catalyzes the conversion of ATP to cAMP (Peterkofsky et al., 1993). By comparison, the *M. tuberculosis* H37Rv genome contains 15 class III adenylate cyclases (McCue, McDonough & Lawrence, 2000), 10 of which have confirmed, biochemically distinct, activity (Castro et al., 2005; Linder, Hammer & Schultz, 2004; Linder, Schultz & Schultz, 2002; Reddy et al., 2001; Abdel Motaal et al., 2006; Cann et al., 2003; Shenoy & Visweswariah, 2006; Guo et al., 2001; Sinha et al., 2005; Tews et al., 2005). Classes of adenylate cyclases contain separate, unrelated gene families with no structural similarities. Class I cyclases are composed of cytoplasmic cyclases found in enterobacteria, class II cyclases are secreted toxins, while class III is the largest and most diverse class. Class III cyclases are typically multidomain proteins which include all of the known eukaryotic cyclases as well as a variety of prokaryotic cyclases (Danchin, 1993). *M. tuberculosis* class III cyclases include receptor, membrane bound, and soluble type family members, and two (Rv1625c and Rv2435c) belong to the mammalian type adenylyl cyclase grouping (McCue, McDonough & Lawrence, 2000; Shenoy & Visweswariah, 2006; Reddy et al., 2001). This abundance and diversity of adenylate cyclases suggests a complex role for cAMP signaling in *M. tuberculosis*. Besides *M. tuberculosis*, other mycobacterial species, including the nonpathogenic *M. smegmatis*, actinobacteria, alphaproteobacteria, and cyanobacteria also contain a wide diversity of annotated class III adenylate cyclases signifying that the *E. coli* paradigm is not transferrable to all bacterial species (McCue, McDonough & Lawrence, 2000; Shenoy et al., 2004).

With a high number of adenylate cyclases there is likely to be significant redundancy in mycobacterial cAMP production. Enzymatic regulation of adenylate cyclases in response to changing environments has been identified as a regulatory mechanism in *M. tuberculosis* (Abdel Motaal et al., 2006; Linder, Hammer & Schultz, 2004; Cann et al., 2003; Linder, Schultz & Schultz, 2002). However we hypothesize that it is unlikely for all cyclases to be present in the cell at the same time. Instead, it is more probable that cyclase regulation is first controlled at the level of transcription, with expression dependent on specific environmental signals or growth stimuli. For instance, Dass et al. (2008) demonstrated that expression of MSMEG_3780 is downregulated under low pH conditions and that downregulation is tied to decreased production of cAMP in that environment (Dass et al., 2008). While Dass et al. (2008) focused on characterizing the detailed regulation of one cyclase we have examined expression of all eight *M. tuberculosis* cyclase orthologs found in nonpathogenic *M. smegmatis* to determine the role transcriptional regulation may have on the availability of cyclases in the cell.

## Materials and Methods

### Bacterial culture

*M. smegmatis* mc^2^155 was grown in Tryptic Soy Broth (TSB) supplemented with 0.05% Tween-80. Cultures were grown in ambient air or 5% CO_2_ at 37°C in 25 cm^2^ tissue culture flasks rocking with gentle agitation. For gene regulation assays, late log phase cultures were exposed to low pH (TSB adjusted to pH 5.5 with 0.1 M HCl), starvation (incubation in Phosphate Buffered Saline), hydrogen peroxide (5 mM), or nitric oxide (10 mM diethylenetriamine/nitric oxide adduct) for 4 h and gene expression was compared to non-exposed cultures.

### RNA preparation

Late log phase culture of *M. smegmatis* mc^2^155 was pelleted and resuspended in RNase-free water. Cells were mechanically disrupted using a bead beater (BioSpec Products) for four rounds of beating on high for 1 min each, in a mixture of 0.1 mm zirconia-silica beads (BioSpec Products), 45% TRIzol (Invitrogen), 45% acid phenol, and 10% chloroform-isoamyl alcohol (24:1). RNA was precipitated with isopropanol/3 M sodium acetate (pH 5.2) and resuspended in RNase-free water. RNeasy Mini Kit and RNase-free DNAse (Qiagen) were used to remove contaminating DNA following manufacturer’s specifications.

### Semi-quantitative RT-PCR

cDNA was prepared from 0.5 µg of RNA using the iScript cDNA synthesis kit according to the manufacture’s specifications (BioRad). PCR was run using a series of cDNA dilutions (0–1:1000) as templates to ensure reactions chosen for quantitation were in the linear range of the PCR (Table 1 for primers). Reactions were performed at 94°C for 1 min, 57°C for 1 min and 72°C for 1 min followed by a 10 min extension at 72°C. Control reactions were performed against 16S rDNA using cDNA diluted 10^−5^. PCR products were separated on agarose gels and band densities quantified using ImageJ software (Abramoff, Megalhaes & Ram, 2004). The 16S PCR products from all growth conditions were normalized to one another before quantitation of individual genes, to ensure equal levels of starting RNA in each reaction. 16S rDNA PCR was also performed using total RNA without reverse transcription to ensure the absence of DNA contamination.

**Table 1:** Primers used throughout this study.

RT-PCR		Promoter:reporter fusions	
Gene	Sequence^a^	Gene	Sequence
MSMEG_0545	F-GATCGAGCCGAAGAACTGTG	Rv1264	F-NNNNGGATCCGACGATGTCGACGTAGTTGT
	R-ATTGAGGGCGATCAAGTGAG		R-NNNNGGTACCGCGCACGTGGTCTGTCAC
MSMEG_3578	F-CGATCGTCAACAAACTGGTG	Rv1318c	F-NNNNGGATCCAGATGCCCGAGGTCCAAG
	R-CAGGTATCCGTTGTGCAGTG		R-NNNNGGTACCGTGCTCTTGGCCGACAT
MSMEG_3780	F-CATACTCTTGCGCCTGTGAA	Rv1319c	F-NNNNGGTACCCGATCGCGGTCATGTACTC
	R-CCCTGAGGTCTTTCGTGCT		R-NNNNGGTACCTGTGTCGGTCACGCTCTAAG
MSMEG_4279	F-CGACCTGTCGGATTTCACC	Rv1359	F-NNNNGGATCCGGAGGTTCGCCACAAGATT
	R-CATCTGATGCCGCAGAACT		R-NNNNGGTACCCGATACCTTCCGGCTAAGCA
MSMEG_4477	F-AGCCTGGCGTATCAGCTCT	Rv1647	F-NNNNGGATCCAGCGGGAACCGCTAGGG
	R- ACGGTCCAGAACAATTCGAC		R-NNNNGGTACCGTAGGTGGTGCGGGCTGAG
MSMEG_4924	F-GTGACGCTGGAGAACCTGAC	Rv1900c	F-NNNNGGATCCACCGGATCGATCACTTGC
	R-AAGATGAAGCCGAACACCAG		R-NNNNGGTACCATGGTCGAGGCGGATCAC
MSMEG_5018	F-ATCCAGCCACTCCTGGAAG	Rv2212	F-NNNNGGATCCGCAGATTGGTGATGCTCAGA
	R-TGAGCAGCCAGTTGATCAGT		R-NNNNGGTACCGACCATAGCAGGACGTCACC
MSMEG_6154	F-CCTGCTCAACGAGTTCTTCC	Rv2435c	F-NNNNGGATCCGTCTGAGTGCGTCGTCGTT
	R-GCGTCACCCTGGAACTTGT		R-NNNNGGTACCTACCGAGTCCAGTGCCTCAC
16S rDNA	F-GCGATACGGGCAGACTAGAG	Rv3645	F-NNNNGGATCCAATCACCACGATCTGCCAGT
	R-CCTCCTCCTGATATCTGCGCATT		R-NNNNGGTACCGCATGCTCAGCGAGAACAG
*sig*A	F-TCGAGGACGAGGAAGAAGAA	*tuf*	F-NNNNGGATCCGTGCGGAAGTAGAACTGCGG
	R-CCTCCAGCAGATGGTTTTTG		R-NNNNGGTACCAGGAAGTTGAGATCGTCGGC

**Notes.**

a F, forward primer; R, reverse primer.

NNNN, added nucleotides to aid in restriction digestion, sequence irrelevant.

### Gene reporter construction and assay

Promoter:*gfp* reporter strains were generated for gene expression analysis of *M. tuberculosis* promoter regions in a *M. smegmatis* background. The intergeneic DNA sequences of the adenylate cyclase genes were amplified by PCR (Table 1 for primer sequences) and amplified DNA was cloned into pGFPoriM, which carries a promoterless *gfpmut2* gene as previously described (Purkayastha, McCue & McDonough, 2002; Florczyk et al., 2003). Constructed plasmids were electroporated into *M. smegmatis* mc^2^155 at 2500 mV (Eppendorf 2510 Electroporator). GFP fluorescence from cultured cells was detected using GloMax Multi + Detection System (Promega) and normalized to 10^6^ bacteria based on OD_600_.

## Results and Discussion

### Regulation of *M. smegmatis* adenylate cyclases

The genome of *M. smegmatis* contains ten annotated adenylate cyclases, eight of which have orthologs in pathogenic *M. tuberculosis* (Kapopoulou, Lew & Cole, 2011). In order to determine the role of gene expression in adenylate cyclase availability we systematically examined transcription of all eight orthologs using semi-quantitative RT-PCR. Gene expression was examined with a focus on *M. tuberculosis* orthologs, using a variety of environments known to be relevant for *M. tuberculosis* infection. Conditions examined include starvation, low pH, oxidative and nitrosative stress and 5% CO_2_. Evidence suggests that non-growing persistent *M. tuberculosis* is exposed to nutrient starvation in lung granulomas (Betts et al., 2002; Nyka, 1974) while low pH, oxidative and nitrosative environments are known to occur following macrophage phagocytosis of *M. tuberculosis* (Smith, 2003; Liao, Fan & Bao, 2013; Chan et al., 1992).

**Figure 1:**
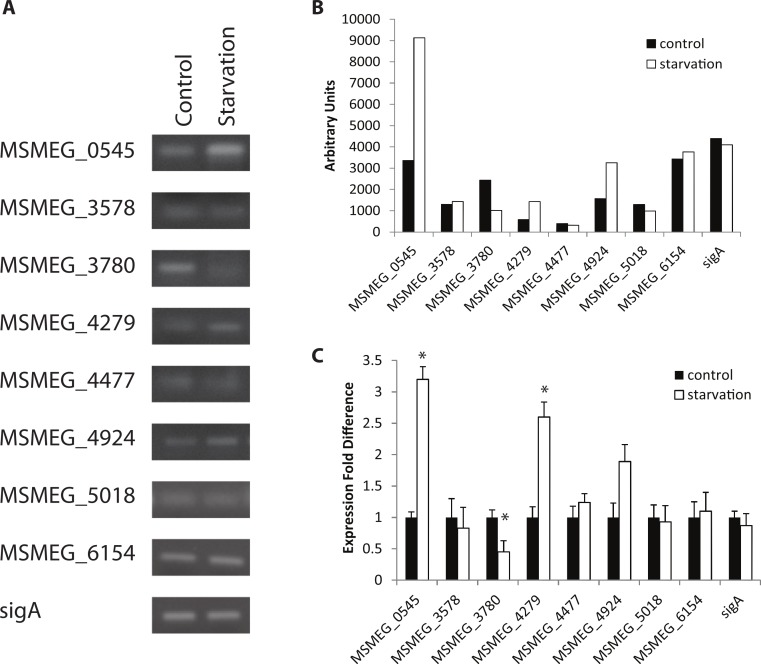
Regulation of *M. smegmatis* adenylate cyclase genes under starvation conditions. Semi-quantitative RT-PCR was used to compare adenylate cyclase mRNA levels between late-log phase cultures incubated for 4 h in mycomedia (control) or PBS (starvation). (A) A representative depiction of PCR-amplified cDNA separated using agarose gel electrophoresis. (B) Quantification of control (black bars) and starvation (white bars) PCR products depicted in A. (C) Average of three different experiments represented as fold differences of starvation compared to control expression. ∗ indicates statistically significant difference between expression in control and starvation for an individual gene (*P* < 0.05.)

**Figure 2:**
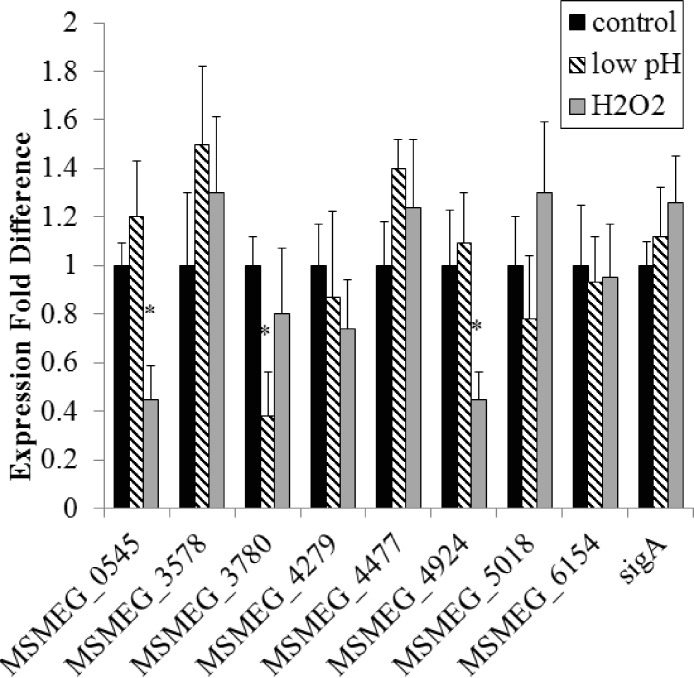
Regulation of *M. smegmatis* adenylate cyclase genes under low pH and oxidative stress. Semi-quantitative RT-PCR was used to compare adenylate cyclase mRNA levels between late-log phase cultures incubated for 4 h in mycomedia (control, black bars), mycomedia adjusted to pH 5.5 (low pH, hatched bars), and mycomedia containing H_2_O_2_ (H_2_O_2_, grey bars). Results are the average of three independent experiments and are expressed in fold difference comparing experimental condition to control. ∗ indicates conditions with statistically significant differences in expression compared to control for an individual gene (*P* < 0.05).

Expression under control conditions (growth in TSB + Tween-80) indicated that all eight adenylate cyclase genes are transcribed at the same time in the cell, albeit with varied levels of transcription (Fig. 1). Out of all conditions tested, starvation and oxidative stress (H_2_O_2_ exposure) affect gene expression the most, with three genes showing statistically significant changes in expression under starvation conditions and two demonstrating statistical changes in expression under oxidative stress. This enhances the evidence of cAMP-mediated gene regulation in nutrient-limiting conditions as 30% of the *M. tuberculosis* CRP_Mt_ regulon has previously been linked to nutrient starvation (Bai, McCue & McDonough, 2005). Two genes (MSMEG_0545 and MSMEG_4279) were upregulated 2–3 fold after 4 h of starvation while one gene (MSMEG_3780) was observed to be downregulated approximately 2 fold after starvation and exposure to low pH. Additionally, two genes (MSMEG_0545 and MSMEG_4924) were downregulated approximately 2 fold under oxidative stress (Figs. 1 and 2). No expression differences were seen during nitrosative stress or the presence of CO_2_ (data not shown).

Interestingly, the expression of only four of the eight adenylate cyclases was influenced by the environments examined, and those observed expression changes, though significant, were not dramatic. Combined with the result that all eight genes were transcribed at various levels in all environments tested we conclude that our results counter our hypothesis and indicate that transcriptional regulation plays only a minor role in controlling the availability of various adenylate cyclases in the cell. Three of the four genes with environmentally-altered expression encode for soluble adenylate cyclase proteins suggesting that transcriptional regulation may play a larger role in the availability of soluble cyclases as opposed to the membrane associated and multi-domain structures. It is likely that biochemical regulation of various protein domains has the dominant role in regulation of cAMP production by the redundant cyclases.

### Regulation of *M. tuberculosis* adenylate cyclases

**Figure 3:**
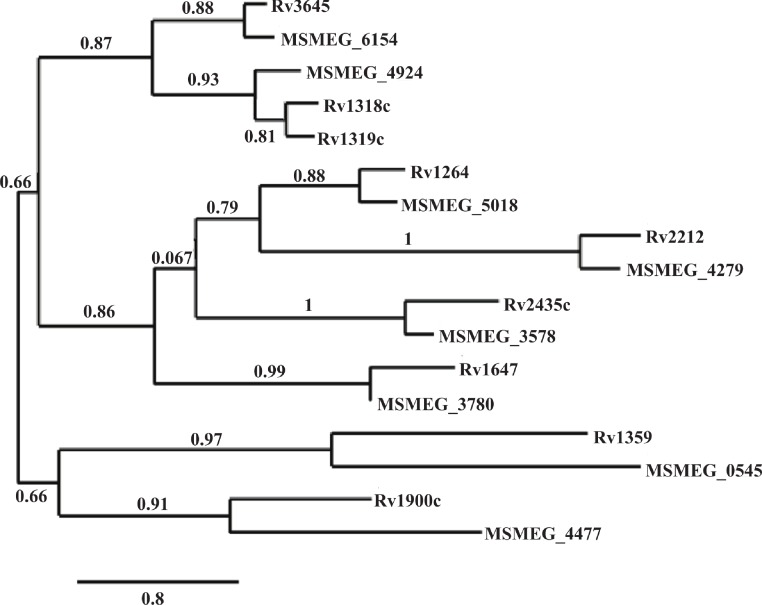
Phylogenic analysis of *M. tuberculosis* and *M. smegmatis* adenylate cyclase orthologs. Amino acid sequences obtain from MycoBrowser databases were aligned and used to generate a phylogenic tree using Phylogeny.fr, combining alignment with MUSCLE, tree building with PhyML, and TreeDyn for tree rendering. The percentage of replicate trees in which the associated orthologs clustered together in the bootstrap test is shown above the horizontal branches. The branch lengths are drawn to represent evolutionary distances.

Of the ten annotated *M. smegmatis* cyclases, eight have reported orthologs in *M. tuberculosis* and *M. bovis*, seven in *M. marinum*, and five in *M. avium* (Kapopoulou, Lew & Cole, 2011). Phylogenic analysis based on protein sequence alignment was completed using Phylogeny.fr, online software that combines MUSCLE for multiple alignment, PhyML for tree building, and TreeDyn for tree rendering (Dereeper et al., 2008). This analysis confirmed the similarities of the *M. tuberculosis* and *M. smegmatis* ortholgos (Fig. 3). Additionally, promoter regions of each ortholog pair, represented by the 500 nucleotides upstream of the putative ATG start site of each gene, were compared for sequence similarities using the sequence alignment program T-Coffee (Notredame, Higgins & Heringa, 2000; McWilliam et al., 2013). Percent identities ranged from 51.94% to 82.08% (Table 2), indicating enough similarity to predict regulation would be similar between orthologs.

**Table 2:** Percent identity between Mtb and *M. smegmatis* ortholog promoters.^a^

MSMEG gene	H37Rv gene	Percent identity^b^
0545	Rv1359	57.02
3578	Rv2435	46.3
3780	Rv1647	51.94
4279	Rv2212	78.97
4477	Rv1900	54.42
4924	Rv1318c	54.6
4924	Rv1319c/ Rv1320c	82.08
5018	Rv1264	54.01
6154	Rv2645	69.28

**Notes.**

a Promoter region defined as the 500 nucleotides upstream of translation start site.

b Determined with T-Coffee multi-sequence alignment.

**Figure 4:**
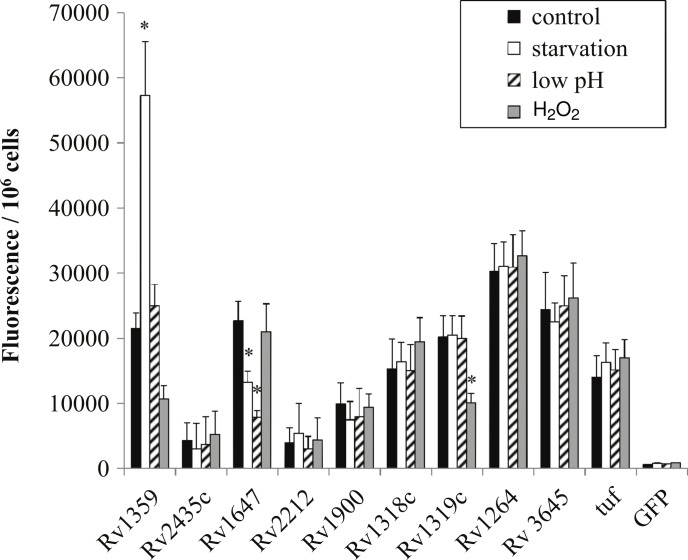
Regulation of *M. tuberculosis* adenylate cyclase genes. GFP: promoter fusions containing *M. tuberculosis* promoters were electroporated into *M. smegmatis* and used to examine adenylate cyclase expression in late log phase cultures after 4 h incubation in mycomedia (control, black bar), PBS (starvation, white bar), mycomedia adjusted to pH 5.5 (low pH, hatched bars), and mycomedia containing H_2_O_2_ (H_2_O_2_, grey bars). Fluorescence was normalized to 10^6^ cells and represented as the average of three independent experiments. ∗ indicates conditions with statistically significant differences in expression compared to control for an individual gene (*P* < 0.05).

In order to determine if regulation in *M. smegmatis* was similar to that of the pathogenic orthologs we generated *gfp*:promoter fusions using the intergenic regions amplified from H37Rv chromosomal DNA. Overall, regulation between species orthologs was very similar. Rv1647 showed downregulation in both starvation and low pH similarly to MSMEG_3780, supporting the observation by Dass et al. (2008) who reported comparable regulation (Fig. 4). Additionally, Rv1359 showed similar regulatory patterns of upregulation in starvation and downregulation in oxidative stress as did MSMEG_0545 (Fig. 4). Oxidative stress and starvation are key environments for *M. tuberculosis* infection and latency respectively. Interestingly, Rv1359 is one of five H37Rv annotated adenylate cyclases that has not been shown to be biochemically active. The catalytic mechanism of class III adenylate cyclases is predicted to be a 2-metal ion reaction similar to DNA polymerase, where one metal ion, typically magnesium, associates with ATP and the second is involved in the catalysis reaction (Zimmermann, 1998). The Rv1359 protein sequence contains a glycine residue in place of the first of two required metal binding aspartates, along with missing a critical arginine residue, making it likely that this protein cannot function as an enzymatically active homodimer (Shenoy et al., 2004). Blastp alignment of the orthologs Rv1359 and MSMEG_0545 indicates that MSMEG_0545 is also missing the first aspartate residue, containing an arginine in this location instead, suggesting that MSMEG_0545 may also be catalytically inactive.

Transcriptional regulation of Rv1359 and MSMEG_0545 suggests that these genes do encode functional proteins. Guo et al. (2009) identified interactions between the Rv1359 protein and the promoter regions of rubredoxin encoding genes *rub*A and *rub*B and fatty acid metabolic genes *fad*D26, *fad*E26, and *fad*E27 (Guo et al., 2009). These genes encode proteins that may function in oxidative stress or starvation conditions. Rubredoxins are iron containing proteins involved with electron transfer, which have been shown to be upregulated in phagocytized *M. tuberculosis* (Buchko et al., 2011), an environment known to expose bacterial cells to oxidative stress. FadD26 is a fatty acid synthetase that is downregulated during starvation of *M. tuberculosis* (Betts et al., 2002) while FadE26 and FadE27 are involved in lipid degradation, a process likely to be important for phagocytized mycobacteria which rely on fatty acids for energy in the phagosome (Schnappinger et al., 2003). We hypothesize that Rv1359, and likely MSMEG_0545, are transcriptional regulators which could play roles in mediating a cAMP signal under oxidative stress and starvation. These proteins may function individually or in conjunction with other proteins as *fad*D26 has already been identified as part of the CRP_Mt_ regulon (Rickman et al., 2005).

The last regulatory similarity observed was the oxidative stress regulation of both MSMEG_4924 and the Rv1320c promoter region. MSMEG_4924 has three predicted orthologs in *M. tuberculosis*, Rv1318c, Rv1319c and Rv1320c. Rv1319c and Rv1320c comprise an operon and are both represented by the Rv1320c promoter region. The regulatory similarity of MSMEG_4924 to Rv1319c/Rv1320c and not Rv1318c correlates with the higher percent similarity to the promoter (82.08%) compared to Rv1318c’s promoter (54.6%) (Table 2).

## Conclusions

Mycobacterial species contain a high number of functional adenylate cyclases when compared to *E. coli*, the typical bacterial model system. The high level of cyclase redundancy led us to hypothesize that only specific cyclases would be expressed in the cell under any given condition. However, the results in this study counter that hypothesis, indicating that all 8 *M. smegmatis* cyclases are transcribed in the cell at one time. While cAMP has been shown to be important for *M. tuberculosis* pathogenesis, adenylate cyclase transcriptional regulation does not appear to have a major role in regulating the availability of cyclases in the cell. Observed changes in cyclase expression in response to varying environments was minor but statistical, and changes occurred under conditions physiologically relevant for *M. tuberculosis* infection. Additionally, regulation of expression was conserved between mycobacterial orthologs, validating the use of *M. smegmatis* as a model system for studying the complex mycobacterial adenylate cyclase/cAMP network.
